# Efficacy of Transarterial Chemoembolization Combined With Camrelizumab in the Treatment of Hepatocellular Carcinoma: A Systematic Review and Meta-Analysis

**DOI:** 10.7759/cureus.48673

**Published:** 2023-11-11

**Authors:** Fatema Ali Asgar Tashrifwala, Vikash Kumar Karmani, Ihtisham Haider, Amna Zubia Syeda, Amber Noorani, Muhammad Saqlain Mustafa, Tirth Dave, Hassan Hafeez

**Affiliations:** 1 Department of Research and Discovery, Stamford Health, Stamford, USA; 2 Department of Internal Medicine, Jinnah Sindh Medical University, Karachi, PAK; 3 Department of Internal Medicine, Fauji Foundation Hospital Lahore, Lahore, PAK; 4 Department of Internal Medicine, Ziauddin University, Karachi, PAK; 5 Department of Biochemistry, Jinnah Sindh Medical University, Karachi, PAK; 6 Department of Medicine, Jinnah Sindh Medical University, Karachi, PAK; 7 Department of Internal Medicine, Bukovinian State Medical University, Chernivtsi, UKR; 8 Department of Internal Medicine, Shalamar Medical and Dental College, Lahore, PAK

**Keywords:** camrelizumab, tace, oncology, carcinoma, hepatology

## Abstract

Hepatocellular carcinoma (HCC) is the most common primary cancer of liver tissue and is often caused by chronic liver diseases. The Barcelona Clinic Liver Cancer (BCLC) staging system is commonly used to determine the stage and prognosis of HCC. Transarterial chemoembolization (TACE) is the recommended first-line therapy for intermediate-stage HCC (patients who have asymptomatic, multi-nodular hepatocellular carcinoma). Over the past 10 years, the combination of TACE with immune checkpoint inhibitors, such as Camrelizumab, has shown promising results in treating HCC.

We conducted a systematic review and meta-analysis following PRISMA guidelines. A comprehensive search of PubMed, MEDLINE, Elsevier, Scopus, ATC abstracts, and the Cochrane Central Register of Controlled Trials (CENTRAL) databases was performed to identify relevant studies on the effectiveness of TACE combined with Camrelizumab in the treatment of HCC. Study selection, data extraction, and quality assurance were conducted by independent investigators. From 1023 identified citations, six studies were included in the final analysis.

The combined results of these studies showed a complete response rate of 7.35%, a partial response rate of 37.10%, stable disease in 28.76% of patients, an objective response rate of 46.13%, a disease control rate of 77.19%, and progression-free survival of 6.2 months.

The combination of TACE and Camrelizumab appears to be a safe and effective treatment option for patients with advanced, recurrent, and unresectable HCC. However, the included studies had limitations such as retrospective design and small sample sizes. Further research is needed to validate and expand on these findings.

## Introduction and background

Hepatocellular carcinoma (HCC) is a malignant tumor that arises from the hepatocytes and is the most common primary cancer of liver tissue [1]. It is usually caused by chronic liver diseases such as hepatitis B or C, alcohol abuse, or non-alcoholic fatty liver disease [1]. HCC is often asymptomatic in its early stages, but as it progresses, symptoms such as abdominal pain, weight loss, and jaundice may occur [2]. HCC accounts for 830,000 deaths globally each year as of 2022. HCC is less common in the United States, with 5.2 new cases per 100,000 individuals. The five-year survival rate of localized HCC is 31% and falls to 2% for metastatic HCC. The number of new cases of liver cancer is predicted to increase by 55.0% between 2020 and 2040, with 1.4 million new diagnoses forecast for 2040. An estimated 1.3 million deaths are predicted to occur in 2040, an increase of 56.4% [3]. There are very few systematic reviews of meta-analyses present currently about the effectiveness of transarterial chemoembolization (TACE) combined with Camrelizumab in the treatment of HCC. Therefore, we have conducted a systematic review of meta-analyses, as this will allow us to comprehensively summarize the evidence regarding the effectiveness of TACE plus Camrelizumab in the treatment of HCC, offer a better use of existing evidence, provide guidance/guidelines for clinicians and researchers, and help identify knowledge gaps in the current literature to inform future research in the area. The diagnosis of HCC is based on imaging or biopsy analysis. The radiological findings in CT and MRI scans show nodules larger than 10 mm in cirrhotic livers or in livers of individuals at a high risk of HCC. Also, there may be a pronounced enhancement in contrast uptake during the arterial phase, followed by a subsequent contrast washout during venous or delayed phases; this exhibits an exceptionally high specificity of nearly 100% [4]. Histologically, early HCC is morphologically different from progressed HCC. In the early stages, hypo-vascularity and changes resembling high-grade dysplasia are seen. More advanced HCC is well-differentiated, with a well-defined capsule and microvascular invasion [4]. Immunohistochemical analysis of HSP70, GPC3, glutamine synthetase, and clathrin heavy chain can also be used to confirm a diagnosis of HCC [5,6]. The most used staging system for HCC is the Barcelona Clinic Liver Cancer (BCLC) system, which determines cancer stage and patient prognosis based on tumor burden, liver disease severity, and patient co-morbidity status [4]. BCLC comprises four distinct stages to identify the most suitable candidates for available interventions. The early stage (A) pertains to patients with early asymptomatic tumors that are amenable to radical treatments such as resection, transplantation, or percutaneous therapies. The intermediate stage (B) refers to patients who have asymptomatic, multi-nodular hepatocellular carcinoma (HCC). Advanced-stage (C) patients exhibit symptoms, such as worsening jaundice, pruritus, hepatic encephalopathy, ascites, palpable mass in the upper abdomen, fever, malaise, weight loss, early satiety, abdominal distension, and cachexia, as well as invasive tumoral patterns such as vascular invasion or extra-hepatic spread. Those in stages B and C may receive palliative treatments or participate in phase II studies or randomized controlled trials of new agents. End-stage disease patients (stage D) have an extremely poor prognosis and are recommended only to receive symptomatic treatment [7]. The discussion section gives more insight into the merits and demerits of the transarterial chemoembolization-Camrelizumab (TACE-C) combination vs. TACE therapy alone in the treatment of HCC. We have also considered the results of previous studies done on this topic [8-31].

## Review

Methodology

This study aimed to conduct a systematic review and meta-analysis in compliance with Preferred Reporting Items for Systematic Reviews and Meta-Analyses (PRISMA) guidelines 2020, to evaluate the effectiveness of Camrelizumab and transarterial chemoembolization in hepatocellular carcinoma. A comprehensive search of relevant literature was carried out using multiple databases such as PubMed, MEDLINE, Elsevier, Scopus ATC abstracts, and the Cochrane Central Register of Controlled Trials (CENTRAL), covering the period from the inception of the study to January 31, 2023. The search strategy was developed through collaboration among the authors and incorporated the use of appropriate MeSH terms and keywords related to Camrelizumab, transarterial chemoembolization, and hepatocellular carcinoma. The search string and PubMed search strategy are available in Table 1.

**Table 1:** Advanced detailed search strategy

Search Strategy:
(camrelizumab) AND (transarterial chemoembolization) OR (TACE) AND (hepatocellular carcinoma)
((("camrelizumab"[Supplementary Concept] OR "camrelizumab"[All Fields]) AND (("transarterial"[All Fields] OR "transarterially"[All Fields]) AND ("chemoembolic"[All Fields] OR "chemoembolisation"[All Fields] OR "chemoembolisations"[All Fields] OR "chemoembolism"[All Fields] OR "chemoembolization"[All Fields] OR "chemoembolizations"[All Fields] OR "chemoembolized"[All Fields]))) OR "TACE"[All Fields]) AND ("carcinoma, hepatocellular"[MeSH Terms] OR ("carcinoma"[All Fields] AND "hepatocellular"[All Fields]) OR "hepatocellular carcinoma"[All Fields] OR ("hepatocellular"[All Fields] AND "carcinoma"[All Fields]))
Translations
camrelizumab: "camrelizumab"[Supplementary Concept] OR "camrelizumab"[All Fields]
transarterial: "transarterial"[All Fields] OR "transarterially"[All Fields]
chemoembolization: "chemoembolic"[All Fields] OR "chemoembolisation"[All Fields] OR "chemoembolisations"[All Fields] OR "chemoembolism"[All Fields] OR "chemoembolization"[All Fields] OR "chemoembolizations"[All Fields] OR "chemoembolized"[All Fields]
hepatocellular carcinoma: "carcinoma, hepatocellular"[MeSH Terms] OR ("carcinoma"[All Fields] AND "hepatocellular"[All Fields]) OR "hepatocellular carcinoma"[All Fields] OR ("hepatocellular"[All Fields] AND "carcinoma"[All Fields])

Data Extraction and Quality Assurance

**Figure 1:**
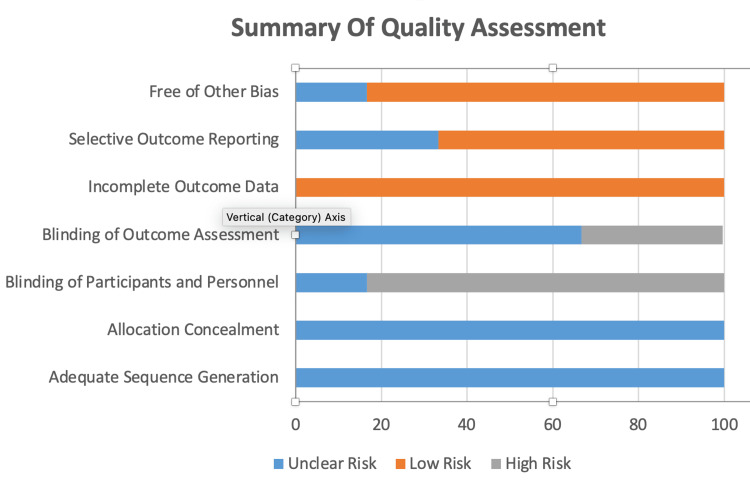
Graphical representation of quality assessment

The data extraction and quality assurance process (Table 2 and Figure 1) involved three separate data extraction team members utilizing established protocols. The accuracy of the data was verified by all three teams, and any discrepancies were addressed through consultation with a fourth team member and the primary investigator. Table 2 and Figure 1 represent the quality assessment of the selected studies.

**Table 2:** Quality assessment The tool used is the Newcastle-Ottawa quality scale (NOS).

Study	Adequate Sequence Generation	Allocation Concealment	Blinding of Participants and Personnel	Blinding of Outcome Assessment	Incomplete Outcome Data	Selective Outcome Reporting	Free of Other Bias
Guo et al [18]	Unclear Risk	Unclear Risk	Unclear Risk	Unclear Risk	Low Risk	Low Risk	Low Risk
You et al [19]	Unclear Risk	Unclear Risk	High Risk	Unclear Risk	Low Risk	Low Risk	Low Risk
JX Zhang et al [22]	Unclear Risk	Unclear Risk	High Risk	Unclear Risk	Low Risk	Low Risk	Low Risk
Ren et al [24]	Unclear Risk	Unclear Risk	High Risk	High Risk	Low Risk	Low Risk	Low Risk
Zhu et al [30]	Unclear Risk	Unclear Risk	High Risk	High Risk	Low Risk	Unclear Risk	Low Risk
Zhang et al [31]	Unclear Risk	Unclear Risk	High Risk	Unclear Risk	Low Risk	Unclear Risk	Unclear Risk

Study Selection Strategy

We conducted a comprehensive search for observational studies (prospective cohort, retrospective cohort, and cross-sectional), case series, and case reports. Exclusions included studies of patients with multiple organ transplants and studies published in non-English languages. Two investigators independently reviewed titles and abstracts of identified citations. A full-text review was performed if the study eligibility could not be determined from the abstract. Discrepancies were resolved by a third independent investigator.

Data Synthesis and Analysis

Patients' demographic parameters were reported as frequencies during the data synthesis and analysis. A random-effects model was used to pool the prevalence estimates and 95% confidence intervals (CI) for each study. The studies' heterogeneity was evaluated using Cochran Q statistics. Several methods were used in the study, including the inverse variance approach, the restricted maximum-likelihood estimator for tau^2, and the Q-Profile method for the confidence interval of tau^2 and tau. The study also provided events per 100 observations to further contextualize the findings. For continuous outcomes, the average methodology was used to provide pooled estimates and 95% confidence intervals. Microsoft Excel was used for analyses, and Open Meta and the R-Studio meta-program were used for meta-analysis. Sensitivity analysis was performed for eight outcomes using the same methodology and software.

**Figure 2:**
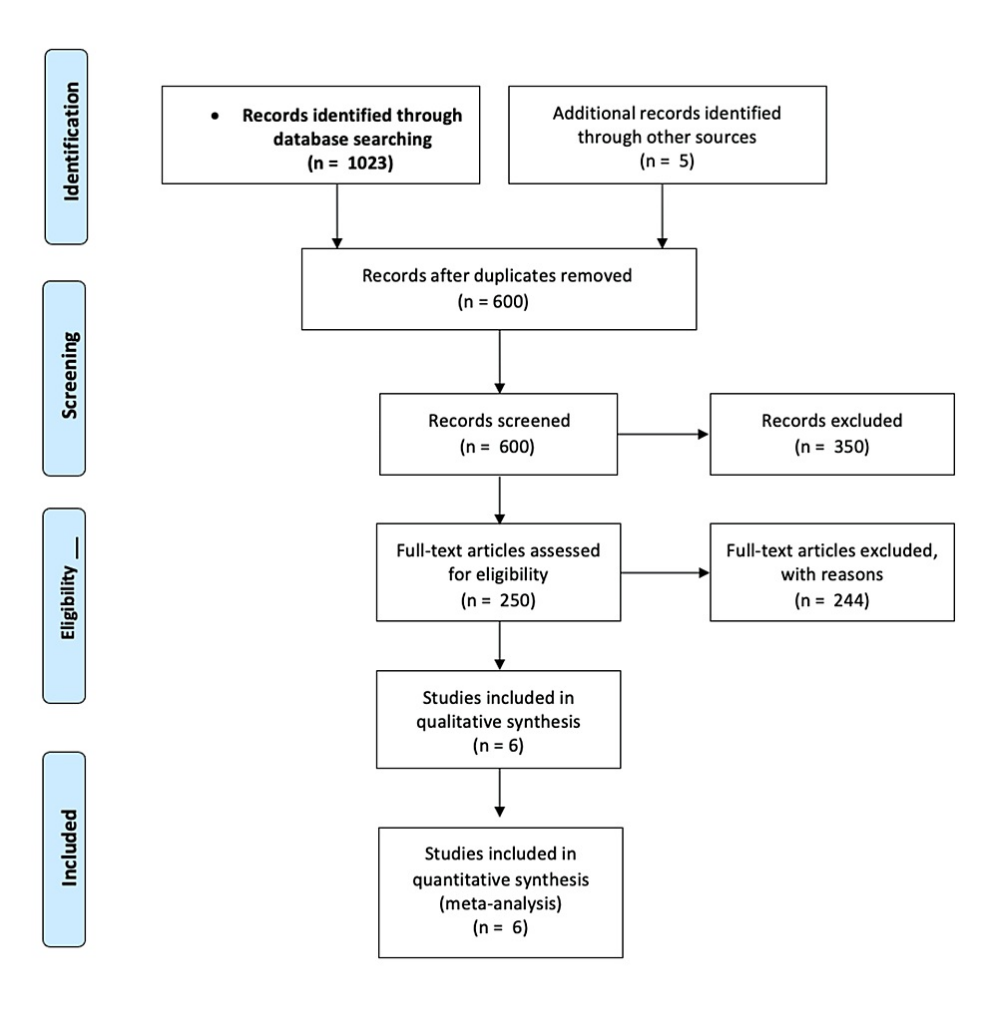
Flow diagram of the assessment of studies identiﬁed by the literature search for inclusion

The meta-analysis was performed on six included studies as shown in Figure 2.

Results

Among the 1023 citations that were retrieved, 250 full-text articles were reviewed, and six were selected to be included in the final study cohort as shown in the PRISMA flow diagram (Figure 2). The objective of the study was to assess the effectiveness of TACE-C in terms of various measures such as complete response, partial response, stable disease, objective response rate, disease control rate, and progression-free survival.

**Figure 3:**
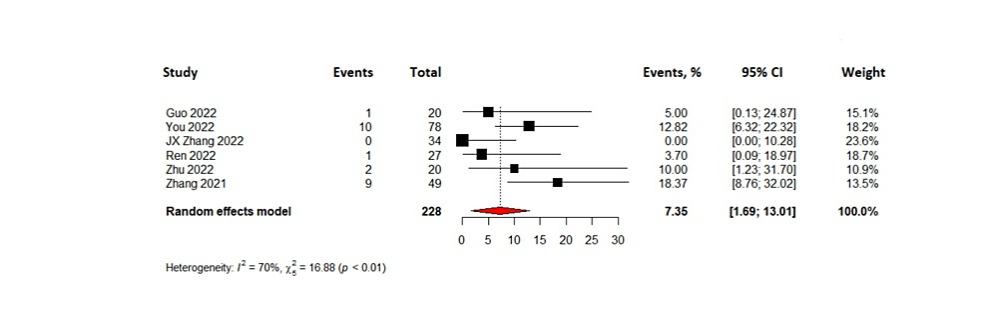
Forest plot of complete response rates in TACE-C for HCC treatment TACE-C: transarterial chemoembolization-Camrelizumab; HCC: hepatocellular carcinoma [18,19,22,24,30,31]

**Figure 4:**
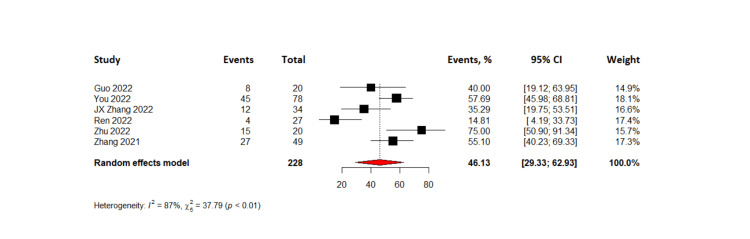
Forest plot of objective response rates in TACE-C for HCC treatment TACE-C: transarterial chemoembolization-Camrelizumab; HCC: hepatocellular carcinoma [18,19,22,24,30,31]

**Figure 5:**
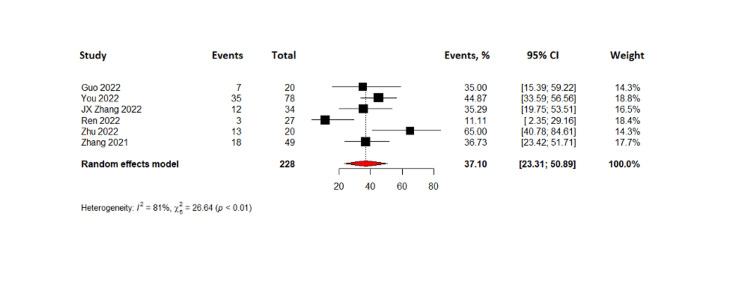
Forest plot of partial response rates in TACE-C for HCC treatment TACE-C: transarterial chemoembolization-Camrelizumab; HCC: hepatocellular carcinoma [18,19,22,24,30,31]

**Figure 6:**
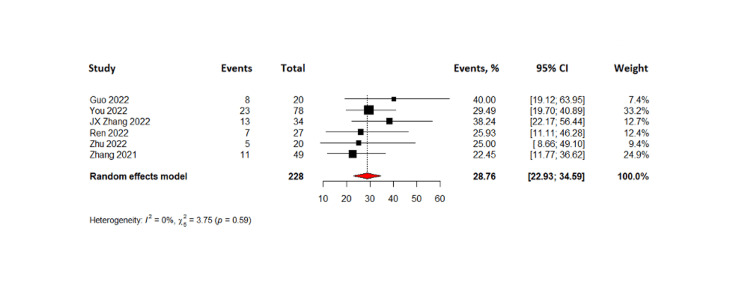
Forest plot of stable disease rates in TACE-C for HCC treatment TACE-C: transarterial chemoembolization-Camrelizumab; HCC: hepatocellular carcinoma [18,19,22,24,30,31]

**Figure 7:**
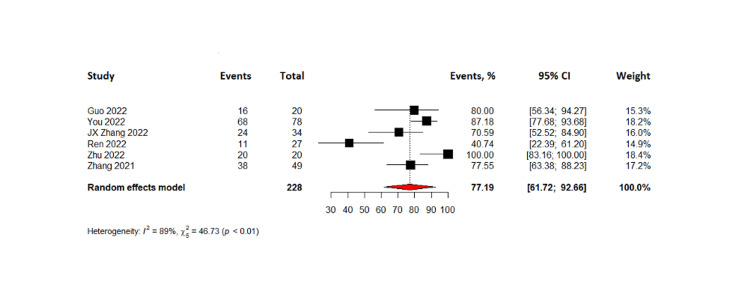
Forest plot of disease control rates in TACE-C for HCC treatment TACE-C: transarterial chemoembolization-Camrelizumab; HCC: hepatocellular carcinoma [18,19,22,24,30,31]

**Figure 8:**
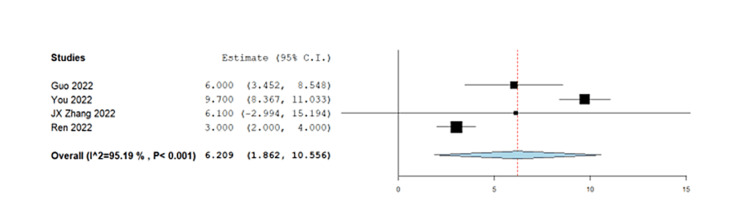
Forest plot of progression-free survival in TACE-C for HCC treatment TACE-C: transarterial chemoembolization-Camrelizumab; HCC: hepatocellular carcinoma [18,19,22,24]

The combined results of 288 patients from six pooled studies revealed that the complete response rate was 7.35% (95% CI= 1.69-13.01%) while partial response was observed in 37.10% with 95% CI (23.31-50.89%). The pooled data also showed that 28.76% of patients had stable disease (95% CI= 22.93 - 34.59) and the objective response rate was found to be 46.13% (95% CI=29.33-62.93%). The disease control rate for all patients in the six studies was found to be 77.19% with 95% CI (61.72 - 92.66%). Lastly, the pooled results from four studies on progression-free survival indicated that TACE-C resulted in progression-free survival of 6.2 months (95% CI= 1.86 - 10.5 months). The forest plots representing this data are shown in Figures 3-8.

Discussion

The combination of transarterial chemoembolization (TACE) and immune checkpoint inhibitors (ICIs) has emerged as a promising treatment option for patients with hepatocellular carcinoma (HCC) [8]. Among these ICIs, Camrelizumab has demonstrated effectiveness and safety when administered in conjunction with TACE for HCC patients. The primary objective of this systematic review and meta-analysis was to assess the efficacy of TACE in combination with Camrelizumab as a treatment approach for HCC. We included six studies in our analysis, which reported the use of TACE along with Camrelizumab in the treatment of advanced, recurrent, and unresectable HCC.

Our analysis found that the combination of TACE and Camrelizumab was generally well-tolerated, with manageable adverse events reported across the studies. The most common adverse events reported were fatigue, fever, and nausea. The assessment of safety is not performed in this analysis, but we are reporting the most known side effects and hence conclude that due to the low risk of serious adverse effects, Camrelizumab is a safe drug to use in most patients in combination with TACE. In terms of efficacy, the combination therapy showed promising results, with a high complete response rate of 7.35%, a partial response rate of 37.10%, stable disease in 28.76% of patients, an objective response rate of 46.13%, a disease control rate of 77.19%, and progression-free survival of 6.2 months combined in all studies. A study by Patidar et al. found that while discussing the safety of TACE alone, the rate of procedure-related adverse events was 15%, including post-embolization syndrome, hepatic decompensation, and vascular complications. With the combination of TACE and sorafenib, the rate of procedure-related adverse events was 10%, with similar complications observed but at a slightly lower frequency. In terms of efficacy, it was found that with the use of TACE alone to treat HCC, the objective response rate (complete and partial response) was 60%, with a median progression-free survival (PFS) of eight months and with the combination of TACE and sorafenib, the objective response rate was 65%, with a median PFS of 10 months [8].

TACE is the recommended first-line therapy for patients with intermediate-stage (B) HCC, according to BCLC criteria [9]. TACE is a locoregional ablation that involves injecting chemotherapy drugs and embolic agents directly into the artery that supplies blood to the tumor, resulting in necrosis and blocking the feeding arteries to cut off the blood supply to the tumor [9]. A study has found that radiofrequency ablation can be combined with TACE for lesions >3 cm because of its high complete ablation rate. A combination of TACE and microwave ablation (MWA) is another popular choice of interventional therapy and is confirmed as effective. Many studies found that the complete ablative rate in the TACE-MWA group was higher than in the TACE-only group [10]. Two randomized controlled trials and two meta-analyses have confirmed that TACE reduces the relative risk of death in comparison to the best supportive care alone and enhances overall survival, with a survival rate of 57% at one year and 26% at three years vs 32% and 3%, respectively, in the control group [11-14]. TACE is not considered a curative procedure, and its efficacy may be limited by tumor angiogenesis of the residual disease [15]. Other treatment options for HCC include liver resection, liver transplantation, percutaneous alcohol ablation, radiofrequency ablation, and radiotherapy [16]. Surgical resection remains one of the main curative options for early HCC in cirrhotic patients and is the treatment of choice in non-cirrhotic patients [17]. In patients with recurrent intrahepatic HCC initially treated with surgical resection, due to factors such as limited functional residual liver, postoperative adhesions, and multi-focal recurrence, re-resection does not present as an option [18]. In these cases, TACE remains the most used treatment for unresectable HCC Fields [19].

Recent articles compare the efficacy and safety characteristics of recombinant human type 5 adenovirus transarterial infusion (H101-TACE) and conventional transarterial chemoembolization (cTACE) in patients with unresectable hepatocellular carcinoma (HCC). A meta-analysis done on the combination of tyrosine kinases and TACE shows promising results for the combination therapy [20,21]. Camrelizumab targets the programmed cell death protein PD-1 and prevents its interactions with its ligands, blocking the antitumor immune response, and resulting in antitumor effects [21]. The studies suggest that combining TACE with Camrelizumab may be a viable treatment option for HCC patients [18,22-27]. A retrospective analysis of 34 HCC patients reported the results two months after intervention with TACE-C indicated that one patient (2.4%) achieved complete response, nine patients (22.0%) achieved partial response, and 15 patients (36.6%) achieved stable disease. Thus, this shows the efficacy of TACE plus Camrelizumab in patients with advanced HCC [24]. A multi-center phase 2 trial conducted in China on 220 patients with a diagnosis of advanced HCC, who had progressed or were intolerant to previous systemic treatment, found Camrelizumab to have significant anti-tumor activity, with a durable response and favorable long survival at 2-year outcomes as well [25]. Other drugs that have been used in combination with TACE for HCC treatment include the multi-targeted tyrosine kinase inhibitors with anti-proliferative and anti-angiogenic effects, lenvatinib, sorafenib, and apatinib [19].

Our findings can be compared with previous studies that have reported the safety and efficacy of TACE plus ICIs in the treatment of HCC. For example, Liu et al. reported the efficacy and safety of TACE plus lenvatinib and Camrelizumab in patients with advanced HCC and found the mean progression-free survival (PFS) to be 11.4 months, whereas, in our study, we reported a PFS of 6.2 months (95% CI= 1.86 - 10.5 months) with the use of TACE-C combination. This difference could indicate that the combination of TACE plus lenvatinib and Camrelizumab is a more effective one, but it is important to note that this was a retrospective study, not a meta-analysis enrolling a sample of 22 patients from March 2018 to December 2019 [28]. Ju et al. conducted a study enrolling 87 patients, exploring the effectiveness and safety of combining TACE with Apatinib and Camrelizumab in treating unresectable HCC. The study found that two patients achieved a complete pathological response, four achieved a major pathological response, and four had a partial response. The median PFS was 10.5 months (95% CI: 7.8-13.1), 10 patients (11.5%) successfully underwent conversion therapy, and all achieved R0 resection. All treatment-related adverse events were tolerated. No serious adverse events were observed, and no treatment-related deaths occurred. They compared this approach to using Apatinib and Camrelizumab alone in a real-world setting. Their findings indicated that combination therapy, involving TACE along with targeted therapy and immunotherapy, led to higher overall survival and better tumor response rates. The synergistic effect of combining TACE, tyrosine kinase inhibitors (TKIs), and Camrelizumab may enhance tumor-fighting effects and enhance overall clinical outcomes for individuals with unresectable HCC [29]. The results of their study demonstrated promising outcomes in terms of antitumor activity and manageable safety when utilizing the combination therapy approach. Collectively, these studies highlight the consistent findings of improved efficacy and manageable safety when combining Camrelizumab with TACE in the management of HCC, both in advanced and unresectable cases.

To sum up, our systematic review and meta-analysis suggest that the combination of TACE and Camrelizumab is a safe and effective treatment option for patients with advanced, recurrent, and unresectable HCC. However, our analysis also identified some limitations that should be considered when interpreting the results. First, the studies included in our analysis were mostly retrospective, which may introduce bias and limit the generalizability of the findings. Second, the sample sizes of the studies were relatively small, which may limit the statistical power of the analysis. Third, the studies included in our analysis were conducted in different populations and settings, which may limit the comparability of the results. Finally, the duration of follow-up varied across the studies, which may affect the accuracy of the survival outcomes reported.

Despite these limitations, our systematic review and meta-analysis provide valuable insights into the safety and efficacy of TACE in combination with Camrelizumab as HCC treatment. Our findings suggest that the combination therapy is generally well-tolerated and shows promising efficacy in treating advanced, recurrent, and unresectable HCC. Future studies should aim to address the limitations identified in our analysis and provide more robust evidence on the safety and efficacy of TACE plus Camrelizumab in the treatment of HCC.

The baseline characteristics of the included studies are represented in Table 3.

**Table 3:** Baseline characteristics The data have been represented as N, %, mean±SD, and standard error (SE). p-value is considered significant if p<0.01.

	Complete Response		Partial Response		Stable		Objective Response Rate		Disease Control Rate		Progression-Free Survival
AUTHOR	TACE-C N(%)	Total (n)	TACE-C N(%)	Total (n)	TACE-C N(%)	Total (n)	TACE-C N(%)	Total (n)	TACE-C N(%)	Total (n)	Standard error SE = (upper limit – lower limit) / 3.92 TAC-C (Mean)
Guo 2022 [18]	1(5%)	20	7(35%)	20	8(40%)	20	8(40%)	20	16(80%)	20	6 months (95%CI: 3.5, 8.6months) SE=1.3
You 2022 [19]	10(12.8%)	78	35(44.9%)	78	23(29.5%)	78	45(57.7%)	78	68(87.2%)	78	9.7 months (95% CI: 7.4 -12.0 months) SE=0.68
JX Zhang 2022 [22]	0(0%)	34	12(35.3%)	34	13(38.2%)	34	12(35.3%)	34	24(73.5%)	34	6.1 months (95%CI: 1.1-19.3 months) SE=4.64
Ren 2022 [24]	1(2.4%)	27	3(11.1%)	27	7(25.9%)	27	4(14.8%)	27	11(40.7%)	27	3 months (95%CI: 2.0, 4.0 months) SE=0.51
Zhu 2022 [30]	2(10%)	20	13(65%)	20	5(25%)	20	15(75%)	20	20(100%)	20	NA
Zhang 2021 [31]	9(18.36%)	49	18(36.73%)	49	11(22.45%)	49	27(55.1%)	49	38(77.55%)	49	NA

## Conclusions

In conclusion, our systematic review and meta-analysis provides compelling evidence supporting the effectiveness and safety of combining transarterial chemoembolization (TACE) with Camrelizumab as a treatment option for patients with advanced, recurrent, and unresectable hepatocellular carcinoma (HCC). We have also compared the safety and efficacy of the treatment options using TACE alone vs. the combination. Although the studies included in our analysis had some limitations, the consistent findings across multiple studies indicate the potential of TACE plus Camrelizumab in managing HCC. Clinicians should consider integrating this combination therapy into their treatment strategies, especially for patients with advanced or unresectable HCC. Further research with larger, prospective studies is warranted to validate and strengthen our findings and to address the identified limitations.
